# Efficacy of Recombinant Methioninase (rMETase) on Recalcitrant Cancer Patient-Derived Orthotopic Xenograft (PDOX) Mouse Models: A Review

**DOI:** 10.3390/cells8050410

**Published:** 2019-05-02

**Authors:** Kei Kawaguchi, Qinghong Han, Shukuan Li, Yuying Tan, Kentaro Igarashi, Takashi Murakami, Michiaki Unno, Robert M. Hoffman

**Affiliations:** 1 AntiCancer, Inc., San Diego, CA 92111, USA; kkawaguchi@surg.med.tohoku.ac.jp (K.K.); qhhan42@gmail.com (Q.H.); li_62@hotmail.com (S.L.); ytan.anticancer@hotmail.com (Y.T.); kenken99004@yahoo.co.jp (K.I.); impressor@hotmail.co.jp (T.M.); 2 Department of Surgery, University of California, San Diego, CA 92093, USA; 3 Department of Surgery, Graduate School of Medicine, Tohoku University, Sendai, Miyagi 9808575, Japan; m_unno@surg.med.tohoku.ac.jp

**Keywords:** recombinant methioninase, methionine dependence, nude mice, orthotopic implantation, patient-derived tumor

## Abstract

An excessive requirement for methionine (MET), termed MET dependence, appears to be a general metabolic defect in cancer and has been shown to be a very effective therapeutic target. MET restriction (MR) has inhibited the growth of all major cancer types by selectively arresting cancer cells in the late-S/G_2_ phase, when they also become highly sensitive to cytotoxic agents. Recombinant methioninase (rMETase) has been developed to effect MR. The present review describes the efficacy of rMETase on patient-derived orthotopic xenograft (PDOX) models of recalcitrant cancer, including the surprising result that rMETase administrated orally can be highly effective.

## 1. Introduction

### 1.1. Methionine (MET)

**Figure 1:**
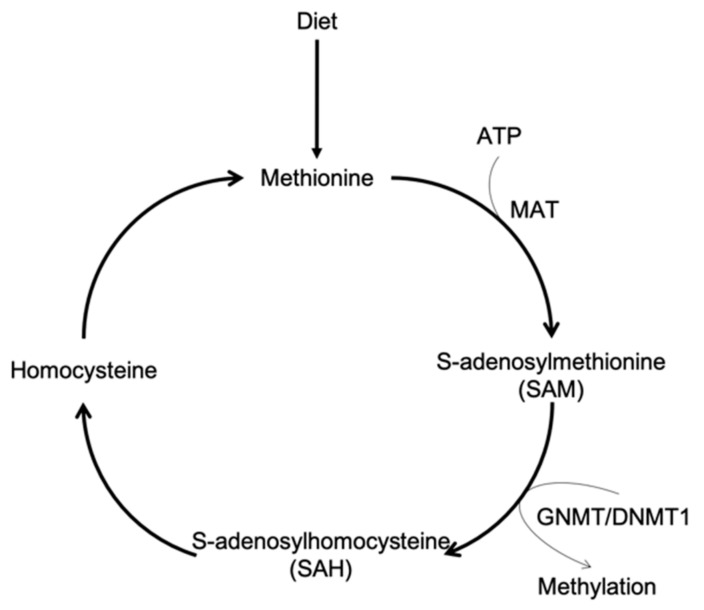
Schema of methionine (MET) metabolism.

Methionine (MET) is an essential amino acid, which is absorbed in the small intestine. The absorbed methionine is used for protein synthesis and converted to S-adenosylmethionine (SAM), which plays an important role in DNA methylation and metabolic reactions. SAM is converted to S-adenosylhomocysteine (SAH) during the methylation of DNA, various proteins and other molecules (Figure 1) [1].

### 1.2. MET Dependence in Cancer

In 1959, Sugimura et al. observed that rat tumor growth was slowed by a MET-restricted (MR) diet [2]. In 1973, it was observed that L5178Y mouse leukemia cells in culture required very high levels of MET to proliferate [3]. Subsequently, most cancer cell lines were found to be MET dependent [4,5]. These cell lines were derived from various cancer types including liver, pancreatic ovarian, submaxillary, brain, lung, bladder, prostate, breast, kidney, cervical, colon, fibrosarcoma, osteosarcoma, rhabdomyosarcoma, leiomyosarcoma, neuroblastoma, glioblastoma and melanoma. Normal unestablished cell strains, thus far characterized, grow well in MET-depleted medium. The very frequent occurrence of MET dependence among these diverse cancer types suggested that MET dependence may be a general phenomenon in cancer and thus an important target for cancer treatment [4].

MET dependence is due not to a deficit in MET synthesis in cancer cells [6] but to elevated MET utilization for aberrant methylation reactions [7,8]. The overuse of MET by cancer cells is termed the Hoffman effect, analogous to the Warburg effect of overutilization of glucose by cancer cells [9]. The Hoffman effect is observed in the clinic in [^11^C] MET-postiron emission tomography (PET) imaging, which gives a stronger signal than [^18^F] fluorodeoxyglucose (FDG) PET, thus indicating that the Hoffman effect is more pronounced than the Warburg effect [10].

### 1.3. Recombinant Methioninase (rMETase)

The enzyme L-methionine-α-amino-γ-mercaptoethane lyase, termed methioninase (METase), was developed to lower the MET level in vivo. METase, was initially purified from *Clostridium sporogene* and catabolized MET to α-ketobutyrate, methanethiol and ammonia [11]. METase suppressed the Walker-256 sarcoma tumor growing in rats more effectively than a MET-free diet [12]. Later, a more stable METase was cloned and purified from *Pseudomonas putida* [13]. METase purified from *P. putida* inhibited the growth of Yoshida sarcoma and lung cancer cells without any overt toxicity, such as body weight loss [14]. Our laboratory cloned and over-expressed the *P. putida* METase gene in *Escherichia coli*, producing high yields of recombinant methioninase (rMETase) [15]. rMETase was reported to have a broad selective efficacy for many cancer cell lines [5].

### 1.4. The Patient-Derived Orthotopic Xenograft (PDOX) Mouse Model

The transplantation of patient-derived tumors to mouse orthotopic sites can replicate the clinical pattern of metastasis [16]. Our laboratory pioneered the patient-derived orthotopic xenograft (PDOX) nude mouse model with the technique of surgical orthotopic implantation (SOI). PDOX models were established from patients with colon [17,18,19], stomach [20], pancreas [21,22,23,24,25,26], breast [27], ovarian [28], lung [29], cervical [30], skin (melanoma) [31,32,33,34,35], bone and soft tissue sarcoma [36,37,38,39,40,41,42].

## 2. Materials and Methods

### 2.1. Mice

Athymic *nu/nu* nude mice (AntiCancer Inc., San Diego, CA, USA), 4–6 weeks old, were used. The mice were housed in a barrier facility on a high-efficacy particulate arrestance (HEPA)-filtered rack under standard conditions of 12 hour light/dark cycles. The animals were fed an autoclaved laboratory rodent diet. All animal studies were conducted in accordance with the principles and procedures outlined in the National Institutes of Health Guide for the Care and Use of Animals under Assurance Number A3873-1. All mouse surgical procedures and imaging were performed with the animals anesthetized by subcutaneous injection of a ketamine mixture (0.02 mL solution of 20 mg/kg ketamine, 15.2 mg/kg xylazine, and 0.48 mg/kg acepromazine maleate). The response of animals during surgery was monitored to ensure adequate depth of anesthesia. The animals were observed on a daily basis and humanely sacrificed by CO2 inhalation if they met the following humane endpoint criteria: Severe tumor burden (more than 20 mm in diameter), prostration, significant body weight loss, difficulty breathing, rotational motion or body temperature drop.

### 2.2. Surgical Orthotopic Implantation (SOI)

For the establishment of PDOX model, patient-derived tumor fragments (5 mm^3^) were initially implanted subcutaneously in nude mice. After several weeks, the subcutaneously-implanted tumors grew to more than 10 mm in diameter. The subcutaneously-grown tumors were then harvested and cut into small fragments (3 mm^3^). After nude mice were anesthetized with the ketamine solution described above, single tumor fragments were implanted orthotopically into each original site or organ to establish the PDOX model.

### 2.3. Recombinant Methioninase (rMETase) Production

Recombinant L-methionine α-deamino-γ-mercaptomethane lyase (recombinant methioninase, referred to as rMETase), EC 4.4.1.11, from *Pseudomonas putida* has been previously cloned and was produced in *Escherichia coli* (AntiCancer, Inc., San Diego, CA, USA) and purified as previously described [15].

### 2.4. Preparation and Administration of Salmonella typhimurium A1-R

GFP-expressing *S. typhimurium* A1-R bacteria (AntiCancer, Inc., San Diego, CA, USA) were grown overnight on LB medium (Fisher Sci., Hanover Park, IL, USA) and then diluted 1:10 in LB medium. The bacteria were harvested at late-log phase, washed with PBS, and then diluted in PBS [43,44,45].

## 3. Results and Discussion

### 3.1. Intraperitoneal Injection of rMETase in PDOX Models of Cancer

**Figure 2:**
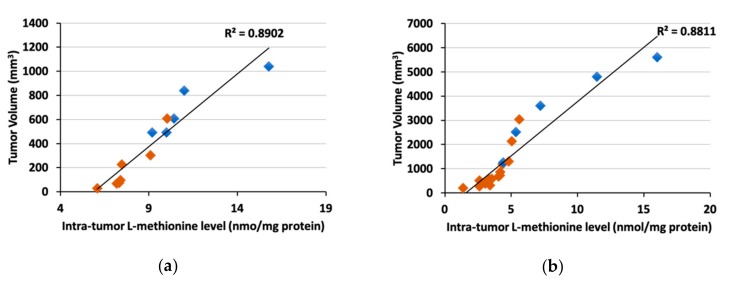
Correlation between tumor volume and methionine (MET) level in pancreatic cancer (**a**) and melanoma (**b**) patient-derived orthotopic xenograft (PDOX). Blue box: Untreated control, red box: Treated with recombinant methioninase (rMETase) [46].

Initially, rMETase was administrated by intraperitoneal injection (i.p.-rMETase) in the PDOX model. i.p.-rMETase was absorbed into the blood circulation through the peritoneum and degraded MET in the blood directly. Kawaguchi et al. demonstrated that intra-tumoral MET levels highly correlated with tumor volume in both pancreatic cancer and melanoma PDOX models, indicating the high degree of MET dependence of the tumors [46]. Furthermore, tumors treated with i.p.-rMETase had a lower concentration of MET and were smaller in size than untreated controls (Figure 2). These results suggested that i.p.-rMETase decreases MET in the blood and suppresses the supply of MET to tumors, thereby inhibiting tumor growth.

**Figure 3:**
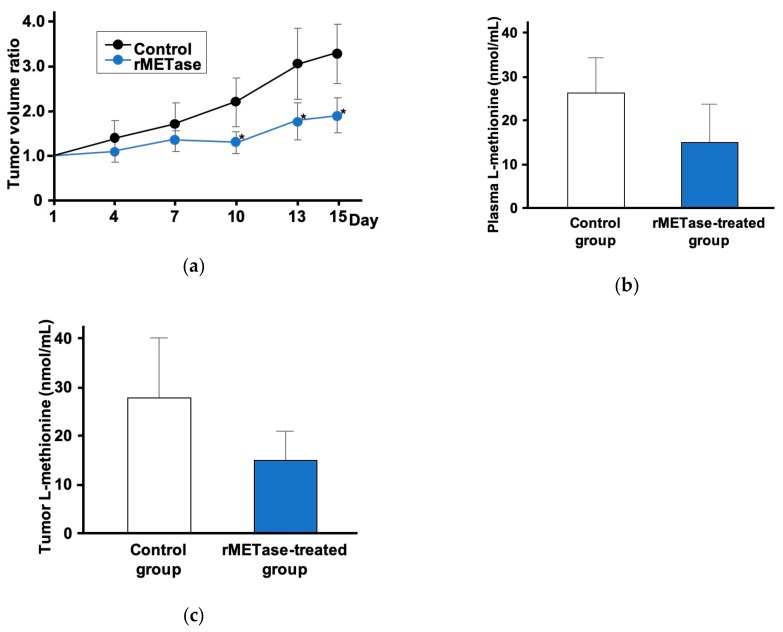
Intraperitoneal (i.p.) recombinant methioninase (i.p.-rMETase) for patient-derived orthotopic xenograft (PDOX). (**a**) Response of Ewing’s sarcoma patient-derived orthotopic xenograft (PDOX) to intraperitoneal injection (i.p.-rMETase). The plasma L-methionine level (**b**) and intra-tumoral L-methionine level (**c**) after i.p.-rMETase treatment. * *p* = <0.05, error bars show the standard deviation (SD) [41].

Our first experience with i.p.-rMETase on a PDOX model was conducted on Ewing’s sarcoma [41]. This study demonstrated that i.p.-rMETase could inhibit tumor growth (Figure 3). Based on this result, other PDOX tumor models were tested with rMETase and high efficacy was shown (Table 1).

### 3.2. Oral administration of rMETase for PDOX

**Figure 4:**
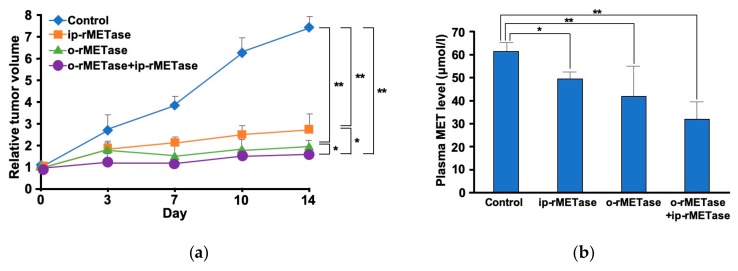
The first report of oral administration of recombinant methioninase (o-rMETase) for melanoma patient-derived orthotopic xenograft (PDOX). (**a**) Comparison of treatment efficacy on oral administration of recombinant methioninase (o-rMETase) and intraperitoneal injection (i.p.-rMETase) for BRAF mutant melanoma PDOX. (**b**) Plasma methionine level treated after recombinant methioninase (rMETase). ** *p* < 0.01, * *p* < 0.05, error bars show the SD [47].

A very surprising result was recently observed that oral administration of rMETase (o-rMETase) was highly effective in a PDOX model. o-rMETase decreased plasma MET concentration and inhibited tumor growth to a greater extent than i.p.-rMETase in a melanoma PDOX (Figure 4) [47]. Subsequent studies showed that o-rMETase could significantly arrest tumor growth in pancreatic cancer PDOX [51]. These are the first reports on oral administration of rMETase. The studies showed that o-rMETase is effective on patient tumors in PDOX models. o-rMETase appears to restrict circulating and tumor MET by degrading MET in the gastrointestinal (GI) tract. Other PDOX studies also demonstrated the usefulness of o-rMETase (Table 1).

### 3.3. Combination of rMETase and Chemotherapy

In a very early study, we used MR in vitro to enhance the efficacy of doxorubicin (DOX) in a co-culture of cancer and normal cells. The MET-dependent cancer cells became blocked in the late S/G_2_ phase by MR. The addition of DOX during MR enhanced its activity as the cells were trapped in S/G_2_, where they are most sensitive to DOX [57]. In a subsequent in vivo study, using fluorescence ubiquitination-based cell cycle indicator (FUCCI)-expressing cancer cells—where color-coded genetic reporters indicate the phase of the cell cycle—rMETase was used to trap cells in S/G_2_ and enhanced the efficacy of the chemotherapy [58,59]. The growth arrest of MET-dependent cancer cells under MR resulted in a reduction in the percentage of mitotic cells in the cell population and the cancer cells were arrested in the S/G_2_ phases of the cell cycle under MR [58,59,60]. The S/G_2_ block by MR is responsible for the high efficacy of the combination of rMETase and chemotherapy.

**Figure 5:**
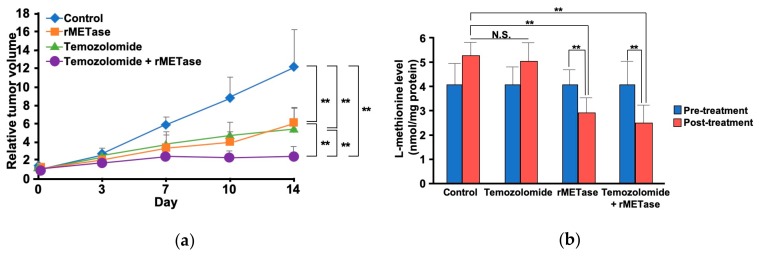
The first report of recombinant methioninase (rMETase) combined with chemotherapy on an orthotopic xenograft (PDOX) model. (**a**) Comparison of treatment. (**b**) Intra-tumoral methionine level after recombinant methioninase (rMETase). ** *p* < 0.01, error bars show the SD [31].

Kawaguchi et al. first reported the rMETase combination with chemotherapy on a melanoma PDOX [31]. Temozolomide (TEM), the first-line chemotherapy for advanced melanoma, and i.p.-rMETase had significantly better efficacy than either therapy alone on a BRAF-V600E mutant melanoma PDOX. The post-treatment L-MET levels in tumors treated with i.p.-rMETase alone, or along with TEM, were significantly decreased compared to untreated controls (Figure 5) [31].

The effectiveness of the combination therapy of rMETase and chemotherapy was also shown in pancreatic cancer [50] and several types of sarcoma [52,53,54,55,56] in addition to BRAF-wild melanoma [48,49] (Table 1).

### 3.4. Combination Therapy of rMETase and Bacterial Therapy

Our laboratory developed *Salmonella typhimurium* A1-R (*S. typhimurium* A1-R) that is auxotrophic for Leu–Arg, which prevents it from mounting a continuous infection in normal tissues. *S. typhimurium* A1-R inhibited or eradicated primary and metastatic tumors as monotherapy in nude-mouse models of major cancers [61], including prostate [43,45], breast [62,63], lung [64,65], pancreatic [66,67,68,69,70], ovarian [71,72], stomach [73], cervical cancer [74], glioma [75,76], melanoma [77] as well as sarcoma [78,79,80,81,82,83], all of which are highly aggressive tumor models.

*S. typhimurium* A1-R decoyed cancer cells in tumors to cycle from the G_0_/G_1_ to S/G_2_/M phases. When the cancer cells were subsequently treated with rMETase, they were selectively trapped in S/G_2_. We showed using sequential treatment of tumors with *S. typhimurium* A1-R to decoy quiescent cancer cells to cycle and rMETase to selectively trap the decoyed cancer cells in the S/G_2_ phase, that subsequent chemotherapy could eradicate tumors in mouse models of human stomach cancer and a metastasis osteosarcoma PDOX model. These results demonstrated a new paradigm of “decoy, trap and shoot (kill)” chemotherapy [52].

**Figure 6:**
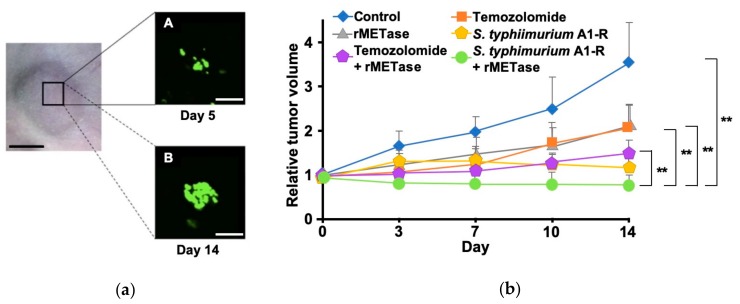
Combination therapy oral administration of recombinant methioninase (o-rMETase) and *S. typhimurium* A1-R. (**a**) Fluorescence imaging of *S. typhimurium* A1-R-GFP cultured from the melanoma patient-derived orthotopic xenograft (PDOX). (**b**) Comparison of treatment. ** *p* < 0.01, error bars show the SD. Obtained permission from [49].

Igarashi et al. first reported the i.p.-rMETase combination with *S. typhimurium* A1-R on an osteosarcoma cisplatinum-resistant lung metastasis PDOX model [52]. They showed that the combination of i.p.-rMETase and *S. typhimurium* A1-R could inhibit tumor growth significantly greater than either monotherapy on an osteosarcoma lung-metastasis PDOX. Another study reported that the combination of o-rMETase and *S. typhimurium* A1-R was also effective for a melanoma PDOX, as shown in Figure 6 [49]. These results showed that the decoy, trap and kill combination of *S. typhimurium* A1-R, rMETase and chemotherapy should be effective for chemo-resistant recalcitrant cancer.

## 4. Conclusions

Here we reviewed the usefulness of MET restriction (MR) therapy using rMETase on PDOX models. MET dependence may be the only known general metabolic defect in cancer. These results have important clinical implications.

## Figures and Tables

**Table 1:** Recombinant methioninase (rMETase) for patient-derived orthotopic xenograft (PDOX).

Cancer Type	Route	rMETase Combination		Reference
Melanoma (BRAF mutant)	i.p.	Alone	Arrest	[31]
		+ Temozolomide	Regress	
Melanoma (BRAF mutant)	Oral	Alone	Arrest	[47]
		+ i.p.-rMETase	Regress	
Melanoma (BRAF wild)	i.p.	Alone	Arrest	[48]
		+ Temozolomide	Arrest	
Melanoma (BRAF wild)	Oral	Alone	Arrest	[49]
		+ Temozolomide	Arrest	
		+ *S. typhimurium* A1-R	Regress	
Pancreatic cancer	i.p.	Alone	Arrest	[50]
		+ Gemcitabine	Regress	
Pancreatic cancer	Oral	Alone	Arrest	[51]
		+ i.p.-rMETase	Regress	
Osteosarcoma	i.p.	Alone	Arrest	[52]
		+ Cisplatinum	Arrest	
		+ *S. typhimurium* A1-R	Arrest	
		+ Cisplatinum+ *S. typhimurium* A1-R	Arrest	
Synovial sarcoma	i.p.	Alone	Arrest	[53]
		+ Doxorubicin	Arrest	
Synovial sarcoma	Oral	Alone	Arrest	[54]
		+ Caffeine	Arrest	
		+ Doxorubicin + Caffeine	Regress	
Liposarcoma	i.p.	Alone	Arrest	[55]
		+ Palbociclib	Regress	
Spindle-cell sarcoma	i.p.	Alone	Arrest	[40]
Spindle-cell sarcoma	i.p.	Alone	Arrest	[56]
		+ Doxorubicin	Regress	
Ewing’s sarcoma	i.p.	Alone	Arrest	[41]
Ewing’s sarcoma	Oral	Alone	Arrest	[42]
		+ *S. typhimurium* A1-R	Regress	
